# New Appliances & Things Medical

**Published:** 1909-08-14

**Authors:** 


### NEW APPLIANCES & THINGS MEDICAL.

[We shall be glad to receive at our Office, 28 & 29 Southampton Street, Strand, London, W.C., from the manufacturers, specimens of all new preparations and appliances.]

### MEUX'S ORIGINAL LONDON STOUT.

(Meux's Brewery, Tottenham Court Road, W.)

We have received two samples of this stout. We have so recently in The Hospital published the report of an exhaustive inquiry in the chemical properties and therapeutic action of beers and stout that we only purpose entering very cursorily into the matter in this notice. In an interesting series of advertisement articles upon Meux's stout, the *Times* some three years ago made the statement that few people can say why ale is yellow and stout is black. Stout is black because part of the malt from which it is made is roasted, and this roasted malt gives to stout not only its characteristic colour, but also its sweetish taste. Among the many stouts analysed by our commissioners was Meux's London stout, with the result that they found this stout satisfactory in all respects. The brewery from which this stout comes was founded in 1764 by Richard Meux, and from that date to this it has continued to produce ales and stouts of high quality. The samples of stout sent to us are quite up to the usual standard, and we think this stout, brewed as it is from the best malt and hops by means of a carefully supervised technique, can be relied upon by the medical profession as a sound product.

### EXPORT BROWN STOUT.—IMPERIAL RUSSIAN STOUT (DOCTOR BRAND).

(Brewed and bottled by Barclay, Perkins and Co., London, S.E.)

The connection of the immortal Dr. Johnson with this brewery lends to it a very particular historic interest, although we dread to think what the great sage would say could he see his own portrait on the bottle labels. Of the two samples sent to us, one is export stout. This stout is brewed especially for export, is more heavily hopped, and contains more alcohol than the ordinary stout. It is, however, a thoroughly good stout, and carefully brewed from the best materials. The value of stout as a dietetic and therapeutic agent has recently been very fully dealt with in The Hospital's Commission on Beers and Stout. In this report it was clearly shown that stout was a beverage of good thirst-quenching properties and high nutritive value, and further that it was often exceedingly useful when given at night as a mild hypnotic. In our opinion, too much importance cannot be laid upon the careful dosing of this as of all other alcoholic beverages. One often hears that stout or beer does not agree with this patient or with that, but in our experience careful attention to dose would almost invariably give one the advantages one wants to obtain for one's patient, and will at the same time prevent the appearance of the undesirable effects. Stout is often best taken not with, but between meals. The nutrient material it contains is easily absorbed, and in the quantity taken practically exerts no action upon the digestive processes. Both Barclay's export stout and Imperial Russian stout are products of the highest class, and can confidently be relied upon as such.

